# Construction and validation of a column-line diagram predictive model for the development of preeclampsia in women with twin pregnancies: A retrospective study

**DOI:** 10.1097/MD.0000000000045407

**Published:** 2025-11-07

**Authors:** Yan Lu, Qiongshan Li, Diyun Meng, Lina Mei, Zhongying Ding, Wenwen Li, Hua Chu, Lin Qin

**Affiliations:** a Department of Obstetrics and Gynecology, Huzhou Maternity & Child Health Care Hospital, Huzhou, Zhejiang, PR China; b Department of Internal Medicine, Huzhou Maternity & Child Health Care Hospital, Huzhou, Zhejiang, PR China; c Department of Laboratory, Huzhou Maternity & Child Health Care Hospital, Huzhou, Zhejiang, PR China; d Department of Ultrasound, Huzhou Maternity & Child Health Care Hospital, Huzhou, Zhejiang, PR China.

**Keywords:** columnar graph, predictive model, preeclampsia, twin pregnancy

## Abstract

This study investigates risk factors for the development of preeclampsia (PE) in women with twin pregnancies and constructs and validates a column-line diagram prediction model for clinical decision-making. Records of 70 women with PE and 70 women without PE were selected from twin pregnancies who underwent labor and delivery at Huzhou Maternity and Child Health Care Hospital between September 2021 and June 2023. The cohort was then divided into a training set (98 cases) and a validation set (42 cases) in the ratio of 7:3 using a simple random sampling method. Clinical risk factors, blood biochemical indexes, and uterine artery pulsatility index of all pregnant women were collected to assess the risk of PE. The results were presented as odds ratios (OR) with 90% confidence intervals (CI). Least absolute shrinkage and selection operator regression analysis was used to screen the predictors and establish an optimized, multifactorial logistic regression-based columnar graph model. Distinction, calibration, and clinical utility of the columnar plot model were evaluated by using the receiver operator characteristic curve, calibration plot, and decision curve analysis. Age (OR = 13.39, 95% CI = 2.152–157.0, *P* = .014), prepregnancy body mass index (OR = 5.979, 95% CI = 1.365–34.27, *P* = .027), mode of conception (OR = 3.498, 95% CI = 1.071–12.79, *P* = .045), serum homocysteine cysteine level (OR = 2.079, 95% CI = 1.193–4.005, *P* = .016), serum β-human chorionic gonadotropin level (Log10; OR = 9.984, 95% CI = 1.467–82.77, *P* = .024), uterine artery pulsatility index (per0.1; OR = 1.347, 95% CI = 1.11–1.7, *P* = .005) were independent risk factors for PE (*P* < .05), and the column-line graph prediction model based on the above 6 risk factors had a good discriminatory degree (area under curve value: 0.880, 95% CI = 0.817–0.944 for training set validation, and 0.831, 95% CI = 0.704–0.958 for validation set validation). The calibration curve showed good agreement between the predicted and actual probabilities of the model (*P* > .05), and the decision curve analysis showed that the model had a high net clinical benefit (threshold probability values: >2.5% for the training set, 18% to 75% for the validation set). The column-line diagram model developed in this study can more accurately predict the risk of developing PE in women with twin pregnancies.

## 1. Introduction

Preeclampsia (PE) is a heterogeneous, multi-system progressive disease that affects about 3% to 5% of pregnant women, and can cause severe maternal and fetal complications such as severe hypertension, disseminated intravascular coagulation, fetal growth retardation, HELLP syndrome, and fetal distress.^[1]^ Twin pregnancies are associated with a higher incidence of PE (7–21%), which progresses more rapidly and is more severe.^[2,3]^ In recent years, with the gradual increase in the reproductive age and advances in assisted reproductive technology, the rate of twin pregnancies is on the rise. Therefore, prediction of PE in this population of pregnant women and timely intervention are crucial. However, there is still a lack of targeted and effective screening methods for the prediction of PE, and the predictive performance of published prediction models is not satisfactory and lacks validation. Furthermore, existing models^[4–8]^ have significant limitations, such as small sample size, retrospective study design, not including crucial Doppler or angiogenic markers, not accounting for chorionicity, etc. This study attempts to construct and validate a diagnostic model for columnar graphs by exploring localized predictive indicators, including Doppler and angiogenic and biochemical markers, such as placental growth factor (PLGF), pregnancy-associated plasma protein-A (PAPP-A), homocysteine (HCY), and beta-human chorionic gonadotropin (β-hCG), to provide a reference for clinical decision-making.

## 2. Material and methods

### 2.1. Clinical information

Women with twin pregnancies who gave birth in Huzhou Maternity and Child Health Care Hospital from September 2021 to June 2023 were selected.

According to maternal health management regulations in Zhejiang Province, all pregnant women are required to establish a prenatal health record before 13 weeks of gestation. These records contain key demographic and clinical information needed for this study, including maternal age, smoking history, body mass index (BMI), obstetric history, method of conception, and whether the mother or her sister has a history of PE. The ultrasound indicator Uterine Artery Pulsatility Index (UTPI) used in this study was a part of routine prenatal care. Laboratory indicators were obtained by testing stored blood samples. All data used in this study were collected by members of the research team.

Eligibility criteria were as follows:

*Inclusion criteria:* twin pregnancy and maternal age 18 to 45 years old.
*Exclusion criteria*: pregnant women with any factors such as learning difficulties or severe mental illness that prevented them from giving full informed consent; the presence of uterine anomalies; women undergoing selective fetal reduction; women lost to follow-up midway through the course of the pregnancy; patients with chromosomal and structural abnormalities of the fetus; unexplained miscarriages or stillbirths; and pregnant women who were taking anticoagulant medications such as aspirin or heparin (to avoid interference with the natural progression of PE and to ensure that the predictive model reflects the true biological process).

The diagnostic criteria for PE were adopted from the “Guidelines for Hypertension and Preeclampsia in Pregnancy” published by the American College of Obstetricians and Gynecologists in 2019.^[9]^ Pregnant women with PE were followed up until 12 weeks postpartum, and pregnant women without PE were followed up until 42 days postpartum.

Sample size was estimated using the following 2 methods: the events per variable rule, which recommends at least 10 outcome events per predictor in logistic regression. The EPV rule indicated a need for 60 PE cases to support 6 variables. Based on the PE incidence in twin pregnancies of 15% to 20%, a total sample size was determined as 300 to 400 twin pregnancies. Using the online tool of the model performance and overfitting method,^[10]^ which determined the required sample size of 410 twin pregnancies.

By combining both approaches, 467 eligible twin pregnancies were identified, with 70 PE cases. Using 1:1 matching (based on gestational age and sample collection timing), 140 cases (70 PE, 70 non-PE) were included in the final analysis, satisfying both sample size and methodological rigor for model development.

**Figure 1.**
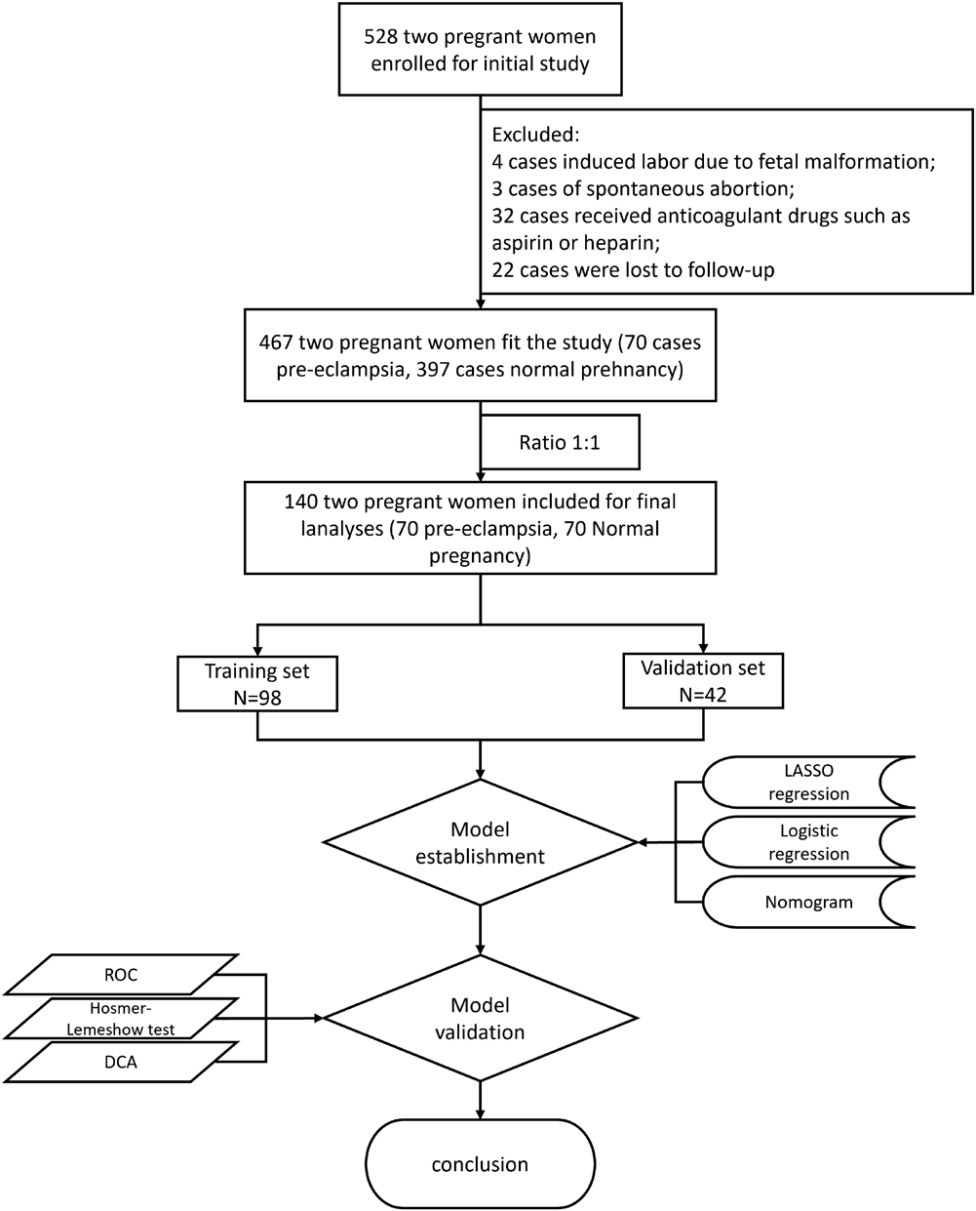
Research pathway diagram. DCA = decision curve analysis, LASSO = least absolute shrinkage and selection operator, ROC = receiver operator characteristic.

Women were divided into the training set (98) and validation set (42) using a simple random sampling method with a ratio of 7:3. The study process is shown in Figure 1.

The clinical research protocol, informed consent, and other filed materials of this study were preapproved by the Ethics Committee of Huzhou Maternal and Child Health Hospital in November 2020 (approval numbers: 2019-r-005, 2020-r-007). The study underwent a follow-up review by the Ethics Committee after the completion of the first subject enrollment intervention.

## 3. Methods

### 3.1. General information collection

The baseline information of the study population, that is, clinical risk factors, including age, smoking history, BMI, maternal history (number of pregnancies, number of deliveries), mode of conception (natural conception/artificial insemination/in-vitro fertilization), and whether the mother or sister had a history of PE, were retrieved from the Zhejiang Maternal and Child Health Care System and the hospital’s electronic medical record system. In this nested case-control study, all pregnant women underwent ultrasonography at 11 + 6 − 13 + 6 weeks of gestation to scan the head and hip length of the fetus to assess the gestational age, to determine the chorionicity and to obtain the UTPI value. Blood samples were collected and preserved at 12 to 22 weeks of gestation.

### 3.2. UTPI

Pregnant women, included in the study, underwent ultrasonography in early pregnancy (11 + 6 − 13 + 6 weeks), and the uterine artery ultrasound parameters were acquired using GE ultrasound instruments Voluson series in 730, E8 and E6 expert and LOGIQ E9 in the negative ultrasound probe 5 to 9 MHz and in the abdominal probe 3.5 MHz. Before the examination, patients were instructed to hold their urine (about 200 mL). During the examination, patients were instructed to lie in the supine position, and the Doppler mode was used to locate the external iliac arteries in the region of the lower uterine segment and then move the probe towards the mid-axis direction. The region of the uterine arteries above the intersection of the uterine arteries with the external iliac vessels up to 1 cm away from the iliac vessels was taken as the sampling window. The direction of the blood flow was angled at <50° with respect to the sampling line, and after the consecutive and regular waveforms of the uterine arteries bilaterally were collected. Three consecutive waveforms were recorded, and the final average of both sides was calculated.

### 3.3. Detection of blood biochemical indicators

A sample (2–4 mL) of peripheral blood was collected from pregnant women in early mid-pregnancy (12–22 weeks), and the supernatant was stored at −80°C. One day prior to testing the sample was allowed to thaw at 4°C. Serum PLGF, PAPP-A, HCY, β-PLGF, and β-HCG were measured using the time-resolved fluorescence immunoassay analyzer 008AS from HITACHI and e801 from Cobas, together with the reagent kits from Ningbo Aocheng Biotechnology Co., based on the manufacturer’s instructions. All operators performing the experimental work were blinded to the clinical information about the study population.

### 3.4. Statistical analyses

SPSS 21.0 software (Huzhou Maternity & Child Health Care Hospital, Huzhou, Zhejiang, China) and RStudio 3.6.3 were used for data processing and statistical analysis. Measured information that conformed to normal distribution with chi-square was expressed as Mean + SD and compared between groups using *t*-test. Non-normally distributed data were expressed as Mean (P25, P75) and compared between groups using Mann–Whitney *U* test. Comparisons of count data were expressed as rates and analyzed using the X2 test or Fisher exact test. Predictors were screened using least absolute shrinkage and selection operator (LASSO) regression analysis, and multifactorial regression analyses were performed using logistic regression analysis with a column-line graph predictive model. The results were reported as odds ratios (OR) with 95% confidence interval (CI), and presented as forest plots. The distinction, calibration and clinical utility of the column chart model were assessed using subject work receiver operator characteristic curves, calibration curve plots and decision curve analysis, and a difference of *P* < .05 was considered statistically significant.

## 4. Results

### 4.1. Baseline characteristics of twin pregnant women in the training and validation sets

A total of 140 women with twin pregnancies were enrolled in this study, including 70 women in the disease group (diagnosed with PE) and 70 women in the control group (no PE). The cohort was then divided into the training set (98) and validation set (42), with no statistically significant difference in the baseline data between the sets (*P* > .05, Table 1).

**Table 1:** Baseline characteristics of the study population.

Variables	Total (n = 140)	Validation (n = 42)	Train (n = 98)	*P*
Group, n (%)				.58
No PE	70 (50)	23 (55)	47 (48)	
PE	70 (50)	19 (45)	51 (52)	
AGE, n (%)				1
<35	122 (87)	37 (88)	85 (87)	
≥35	18 (13)	5 (12)	13 (13)	
BMI, n (%)				.367
18.5 ≤ BMI<25	89 (64)	28 (67)	61 (62)	
<18.5	15 (11)	2 (5)	13 (13)	
≥25	36 (26)	12 (29)	24 (24)	
Pregnancy times, n (%)				.387
0	56 (40)	14 (33)	42 (43)	
≥1	84 (60)	28 (67)	56 (57)	
Parity, n (%)				.099
0	98 (70)	34 (81)	64 (65)	
≥1	42 (30)	8 (19)	34 (35)	
Conception method, n (%)				.384
Nature	72 (51)	18 (43)	54 (55)	
Ovulation	11 (8)	4 (10)	7 (7)	
IVF pregnancy	57 (41)	20 (48)	37 (38)	
Fetal chorionic properties, n (%)				.181
Dichorionic twins	28 (20)	5 (12)	23 (23)	
Monochorionic twins	112 (80)	37 (88)	75 (77)	
Family history of PE, n (%)				1
No	136 (97)	41 (98)	95 (97)	
Yes	4 (3)	1 (2)	3 (3)	
Smoke, n (%)				.316
No	136 (97)	42 (100)	94 (96)	
Yes	4 (3)	0 (0)	4 (4)	
PLGF, median (Q1, Q3; pg/mL)	97.65 (71.25, 149.69)	111.49 (76.7, 162.33)	94.08 (68.49, 141.62)	.132
HCY, median (Q1, Q3; μmol/L)	6.3 (5.6, 7.1)	6.45 (5.85, 7.02)	6 (5.53, 7.1)	.162
β-HCG, median (Q1, Q3; mIU/mL)	55,561.5 (31,752.5, 96,551)	67,381.5 (35,919.75, 94,868.5)	50,795 (31,265.75, 1,00,065.25)	.523
PAPP-A, mean ± SD (μIU/mL)	11,695.99 ± 4826.57	11,289.37 ± 4785.42	11,870.26 ± 4858.07	.514
UTPI, median (Q1, Q3)	1.65 (1.47, 1.8)	1.65 (1.5, 1.81)	1.65 (1.47, 1.8)	.991

β-HCG = beta-human chorionic gonadotropin, BMI = body mass index, HCY = homocysteine, IVF = in-vitro fertilization, PAPP-A = pregnancy associated plasma protein-A, PE = preeclampsia, PLGF = placental growth factor, PLGF = β-human placental growth factor, UTPI = uterine artery pulsatility index

### 4.2. Screening for risk factors of PE in twin pregnancies

**Figure 2.**
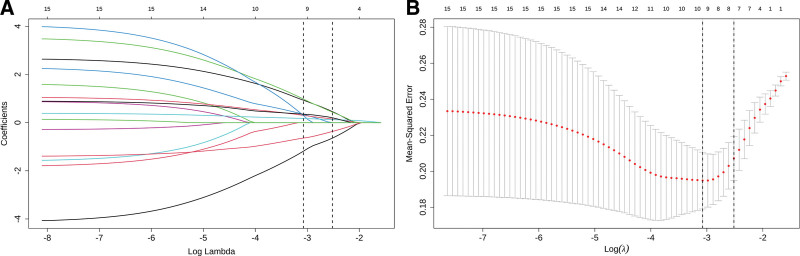
Screening of predictors of twin PE based on LASSO regression. (A) Plot of predictor coefficients, (B) plot of best-matched factors screened by the ten-fold cross-validation method. LASSO = least absolute shrinkage and selection operator, PE = preeclampsia.

A total of 13 variables of clinical risk factors (age, smoking history, BMI, gestational age, number of deliveries, mode of conception, previous history of PE in mother or sister, chorionicity), blood biochemical indexes (PLGF, PAPP-A, β-HCG, HCY), and UTPI in the study population were screened using LASSO regression to find out the risk factors that have a greater effect on the occurrence of PE in twin pregnant women. Ten-fold cross-validation method was selected, and the variable corresponding to Log (λ) with the smallest misclassification error was selected to obtain 7 predictors, namely age, BMI, mode of conception, PLGF, β-HCG, HCY, and UTPI (Fig. 2).

### 4.3. Multifactorial logistic regression analysis of the occurrence of PE in twin pregnancies

Epidemiological surveys have found that the first pregnancy is strongly associated with PE.^[11,12]^ With the occurrence of PE as the dependent variable, 7 predictors screened by LASSO and the number of pregnancies were used as independent variables, and the assignment of values to each of the variables is shown in Table 2. The results of the logistic multifactorial regression analyses showed that advanced age (OR = 13.39, 95% CI: 2.152–157.0), abnormal BMI (OR = 5.979, 95% CI: 1.365–34.27 and OR = 6.435, 95% CI: 1.228–40.98 for overweight and normal BMI, respectively), conception method (OR = 3.498, 95% CI: 1.071–12.79), UTPI (per0.1; OR = 1.347, 95% CI: 1.11–1.7), β-HCG (Log10; OR = 9.984, 95% CI: 1.467–82.77), HCY (OR = 2.079, 95% CI: 1.193–4.005) were all independent risk factors for PE in twin pregnancies (*P* < .05). Advanced age, abnormal BMI, and IVF conception increased the risk of PE in twin pregnancies by about 13, 6, and 3 times, respectively.

**Table 2:** Variable assignment table.

Variable	Assignment
PE	0: No, 1: Yes
Age	1: <35, 2: ≥35
BMI	1: 18.5–25, 2: ≤18.5, 3: ≥25
Pregnancy times	1:0 time, 2: ≥1 times
Conception method	1: Nature, 2: ovulation, 3: test tube baby
PLGF	Normalized by Log10 transformation
HCY	Raw data
β-HCG	Normalized by Log10 transformation
UTPI	per0.1

β-HCG = beta-human chorionic gonadotropin, BMI = body mass index, HCY = homocysteine, PE = preeclampsia, PLGF = placental growth factor, UTPI = uterine artery pulsatility index.

**Figure 3.**
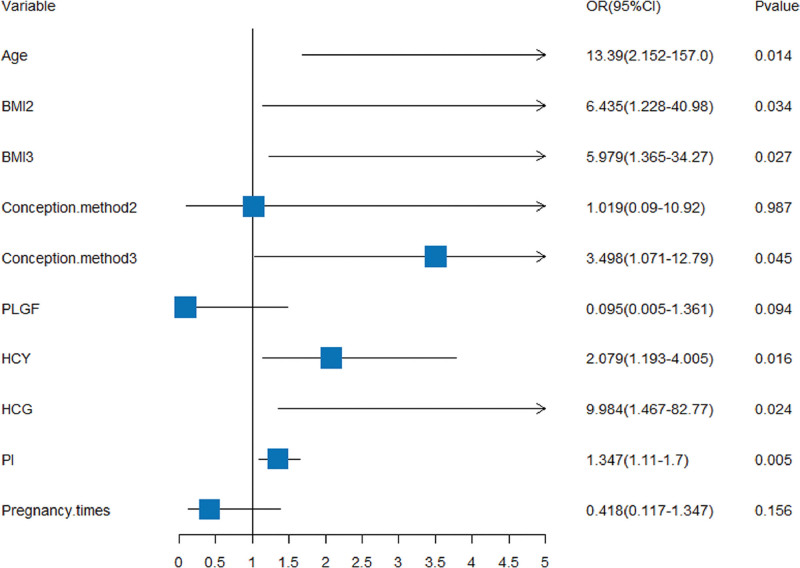
Forest plot of logistic multifactorial regression analysis of risk factors for PE in twin pregnancies. BMI = body mass index, HCG = human chorionic gonadotropin, HCY = homocysteine, PE = preeclampsia, PI = pulsatility index.

The values of β-HCG, HCY, and UTPI in pregnant women with PE were significantly higher than those in normal twin pregnancies. As shown in Figure 3, the prevalence risk of PE increased by about 10 times for every 1 (Log10) conversion of β-HCG, and the prevalence risk of PE increased by about 2 times for every 1 μmol/L of HCY, and by 1.3 times for every 0.1 of UTPI, and the difference was statistically significant (*P* < .05).

### 4.4. Nomogram model construction

**Figure 4.**
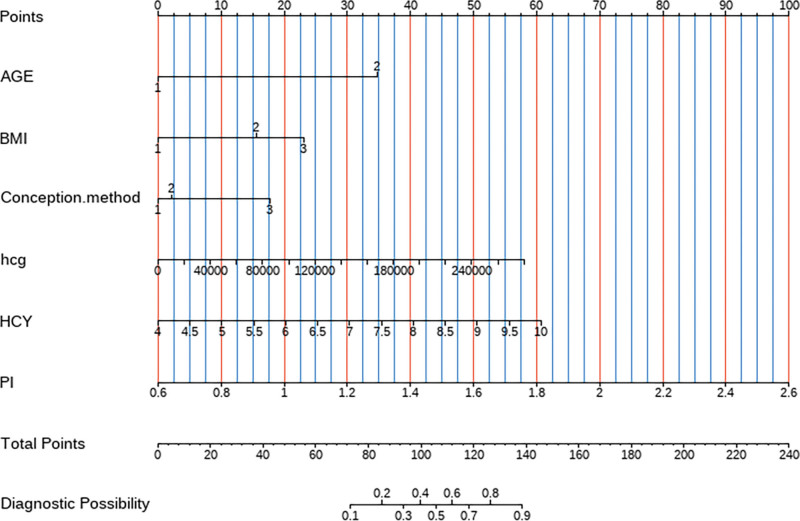
Twin-twin PE nomogram model column-line graphs. Age – 1: <35 years old, 2: ≥35 years old; BMI – 1: 18.5 to 25, 2: <18.5, 3: ≥25; Conception method – 1: Nature, 2: Ovulation, 3: test tube baby. BMI = body mass index, HCG = human chorionic gonadotropin, HCY = homocysteine, PE = preeclampsia, PI = pulsatility index.

The 6 predictors obtained from the multifactorial analysis were used to construct a diagnostic predictive model for twin PE, and the independent variable scores were assigned according to the regression coefficients and displayed in the form of a nomogram (Fig. 4).

### 4.5. Evaluation of the predictive efficacy of nomogram model line charts

**Figure 5.**
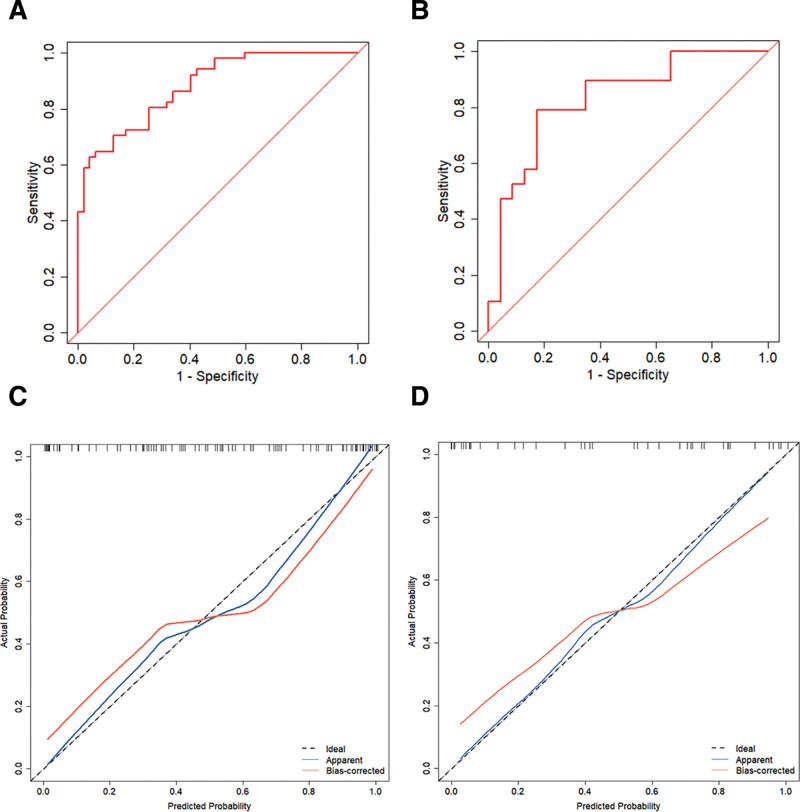
Figure (A, B) shows the ROC of the training and validation sets, respectively; (C, D) shows the calibration curves of the training and validation sets, respectively. ROC = receiver operator characteristic.

Using the analysis of the area under the receiver operator characteristic curve (AUC) of the model, we showed that the AUC of the prediction model in the training set reached 0.880 (95% CI: 0.817–0.944, *P* < .05), with a sensitivity of 0.63 and a specificity of 0.96, and that in the validation set reached 0.831 (95% CI: 0.704–0.958, *P* < .05), with a sensitivity of 0.79 and a specificity of 0.83. These results suggest that the model has good discriminative ability. The calibration curve showed good agreement between the predicted probability and the actual probability of the model (*P* > .05), and the accuracy of prediction was high (Fig. 5).

### 4.6. Evaluation of the clinical utility of the nomogram model column charts

**Figure 6.**
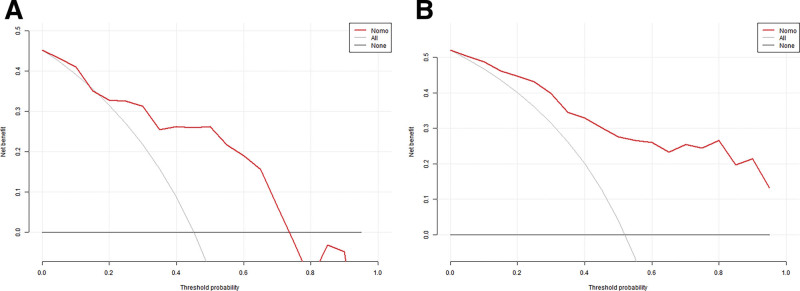
Figures (A, B) the clinical decision curves for the training and validation sets, respectively.

Using clinical decision curves for the analysis, the net benefit of using the column-line graph to predict the risk of PE in twin pregnancies was higher when the threshold probability values in the decision curves of the training set were > 2.5% and the threshold probability values in the decision curves of the validation set were between 18% and 75% (Fig. 6).

## 5. Discussion

Stratified management of PE by screening for maternal risk factors is a common clinical strategy.^[9,11]^ Some studies have shown that maternal risk factors similar to those of singleton PE combined with mode of conception and chorionicity can be used to assess the risk of developing PE in twin pregnancies.^[12,13]^ A study by Fox et al^[14]^ showed that prepregnancy BMI ≥ 25 increased the risk of twin PE by 1.367 times. Taguchi et al^[15]^ found that high BMI, primiparity, family history of hypertension and history of hypertensive disorders in a previous pregnancy were significant risk factors for the development of PE in women with twin pregnancies, with ORs of 1.77 (95% CI: 1.21–2.61), 1.35 (95% CI: 1.08–1.70), 1.50 (95% CI: 1.02–2.17) and 8.85 (95% CI: 2.70–29.0), respectively. A study by Sekhon et al^[16]^ of 4288 twin pregnancies, spanning from 1988 to 2010, found that the incidence of PE in twin pregnancies conceived using in-vitro fertilization (IVF) and those conceived naturally was 13.8% and 7.6%, respectively, with a statistically significant difference. These findings suggest that conception by IVF is an independent risk for PE in twin pregnancies. Risk factors for pregnant women in this group incorporated age, smoking history, BMI, maternal history, mode of conception, previous history of PE in mother or sister and chorionicity.

PE prediction has been a focus of research in recent years. Current studies showed that the best prediction model for single-fetus PE screening combines maternal risk factors, specific biochemical indicators (PLGF, PAPP-A, β-HCG, HCY, vascular endothelial growth factor, soluble tyrosine kinase-1, etc) with uterine arterial ultrasonography and other factors.^[17–19]^ However, only a few studies with small sample sizes have investigated these prediction methods in cases of twin pregnancies with PE, with inconsistent results. Francisco et al^[20]^ found that the combination of maternal risk factors, mean arterial pressure (MAP), UTPI, and PLGF had a good predictive performance (AUC = 0.94, 95% CI: 0.91–0.97) in predicting PE in twin pregnancy, and the detection rates of this model for PE occurring before 32 weeks of gestation, preterm PE, and PE overall were 100%, 69.1%, and 63.4%, respectively. A risk prediction model for PE in twin pregnancies, developed by Maymon et al,^[21]^ incorporated clinical risk factors (MAP, BMI), UTPI, and blood biochemicals of 105 twin pregnancies, and showed a 75% detection rate of PE with an AUC of 0.908 at a false positive rate of 10%. Benkő et al^[8]^ concluded that the efficacy of combined screening in twin pregnancies is not as good as in singleton pregnancies, with a detection rate of 75% for preterm PE (FPR = 10%), which would increase to 40% if the same detection rate were achieved in a population of women with twin pregnancies. Based on existing studies and feasibility, our study chose a combined prediction approach, with the selection of blood biochemical indicators incorporating β-HCG, HCY, PLGF, and PAPP-A4 indicators, and Doppler ultrasonography UTPI values.

The results of our study showed that the prevalence of PE in twin pregnancies was 14.99%, which is consistent with previous reports.^[14,22]^ A nested case-control study method was used to collect general data of pregnant women, test blood biochemical parameters, Doppler ultrasonography for UTPI values, and to screen for risk factors for PE in twin pregnancies by LASSO regression. The results of logistic multifactorial analysis showed that advanced age, abnormal BMI, conception by IVF, high values of β-HCG, HCY and UTPI were independently associated with the development of PE in twin pregnancies, whereas primiparity, history of smoking, previous history of PE in the mother or sister, PLGF, PAPP-A were not associated with the development of PE. The inconsistency between the findings of this study and those of previous reports may be related to a lower smoking rate among women in our country, low level of previous medical care for PE (whether or not the mother or sister had a previous history of PE), different ethnicity of the study population, and the wide selection of the span of gestational weeks for the detection of PLGF and PAPP-A (12–22 weeks). Column-line graph prediction model based on the 6 predictors, used in our study, had good discrimination (AUC value: 0.880 for training set validation, 0.831 for validation set validation), the calibration curves showed good agreement between the predicted probabilities and the actual probabilities of the model (*P* > .05), and the decision curve analysis showed a high net clinical benefit of the model.

In summary, our study is representative of cases at the tertiary-level maternal and child health hospital in eastern China. In this study, the number of PE cases was increased based on the previous study,^[6]^ and PAPP-A, PLGF, and HCY were added as variables, which improved the stability of the model prediction results to a certain extent. The nomogram model column-line diagram was used to make the presentation of the model more intuitive, and internal validation was performed to assess its clinical utility and provide a basis for practical application in the clinic.

Compared with the only 2 existing studies in China that internally validated predictive models for PE in twin pregnancies, the model developed in this study has several significant advantages. Han et al^[5]^ screened clinical risk factors and biochemical indicators, and used a multivariate logistic regression model to identify 10 independent predictors of PE in twin pregnancies, including serum creatinine, uric acid, primiparity, prepregnancy BMI, and routine prenatal care. The model achieved an AUC of 0.7955 in the training set and 0.7868 in the validation set. This study used a different set of predictors and ultimately identified 6 independent risk factors for PE in twin pregnancies. Despite using fewer predictors, the model in this study achieved higher discrimination, with an AUC of 0.880 in the training set and 0.831 in the validation set. A study by Chen et al^[23]^ included a total of 769 twin pregnancies, identifying 27 cases of early-onset PE and 59 cases of late-onset PE. Logistic regression showed that age, BMI, MAP, and PLGF were independent predictors of early-onset PE, while age, BMI, MAP, and PAPP-A were predictors for late-onset PE. The performance of their model was evaluated solely based on discrimination: the AUCs were 0.819 (95% CI: 0.757–0.882) for early-onset PE and 0.661 (95% CI: 0.591–0.731) for late-onset PE. Although the model in this study did not differentiate PE by gestational age at onset, it achieved higher discrimination than the model by Chen et al. Additionally, the current model was evaluated more comprehensively using both calibration curves and clinical decision curves.

In summary, the predictive model for PE in twin pregnancies developed in this study shows strong potential for clinical application and promotion.

This study has some limitations, as it is a single-center study with a relatively small sample size. Women who take anticoagulant drugs were excluded, further reducing the size of the eligible cohort. However, the research shows that the use of aspirin may reduce the incidence of PE and affect the values of UTPI and PLGF.^[24]^ Therefore, participants who were taking anticoagulant drugs such as aspirin or heparin were excluded to prevent potential drug interference with the development of PE. Further prospective cohort studies and multicenter joint studies that would attempt to use new algorithms of machine modeling are needed to improve our ability to predict the risks of developing PE in women with twin pregnancies.

## Author contributions

**Conceptualization:** Yan Lu, Qiongshan Li, Diyun Meng.

**Data curation:** Lina Mei, Zhongying Ding, Wenwen Li, Hua Chu, Lin Qin.

**Formal analysis:** Yan Lu, Qiongshan Li.

**Supervision:** Diyun Meng, Lina Mei.

**Methodology:** Lina Mei, Zhongying Ding, Wenwen Li, Hua Chu, Lin Qin.

**Writing – original draft:** Yan Lu, Diyun Meng, Lina Mei.

**Writing – review & editing:** Diyun Meng, Lina Mei.
